# An Encyclopedic Medical Dictionary

**Published:** 1891-03

**Authors:** 


AN ENCYCLOPEDIC MEDICAL DICTIONARY Vol. II., edited by FRANK P. FOSTER, editor of the *New York Medical Journal.* D. Appleton & Co., New York.

We receive with pleasure the second volume of this great work which well sustains the ambitious purpose of the editor and his able assistants. The book-reviewer is given no opportunity for unfavorable criticism. Of making dictionaries nowadays there seems to be no end, but this immense work for a great many years will be our supreme authority on "medicine and the collateral sciences."

The publishers' task is done faultlessly.

